# Treatment benefit of upfront autologous stem cell transplantation for newly diagnosed multiple myeloma: a systematic review and meta-analysis

**DOI:** 10.1186/s12885-023-10907-1

**Published:** 2023-05-16

**Authors:** Chi-Maw Lin, Lih-Chyun Chang, Wen-Yi Shau, Chi-Ling Chen, Chi-Yuan Yao, Feng-Ming Tien

**Affiliations:** 1 Graduate Institute of Clinical Medicine, College of Medicine, National Taiwan University, Taipei, Taiwan; 2 Department of Otolaryngology, National Taiwan University Hospital, Yun-Lin Branch, Yun-Lin, Taiwan; 3 Division of Pulmonary and Critical Care Medicine, Department of Internal Medicine, National Taiwan University Hospital, Taipei, Taiwan; 4 Division of Hospital Medicine, Department of Internal Medicine, National Taiwan University Hospital, Taipei, Taiwan; 5 Division of Hematology, Department of Internal Medicine, National Taiwan University Hospital, No. 7, Zhongshan S. Rd., Zhongzheng Dist., Taipei City, 100225 Taiwan

**Keywords:** Autologous stem-cell transplantation, Multiple myeloma, Survival, Systematic review, Meta-analysis

## Abstract

**Background:**

Upfront high-dose therapy (HDT) followed by autologous stem cell transplantation (ASCT) remains a profitable strategy for newly diagnosed multiple myeloma (MM) patients in the context of novel agents. However, current knowledge demonstrates a discrepancy between progression-free survival (PFS) and overall survival (OS) benefit with HDT/ASCT.

**Methods:**

We conducted a systematic review and meta-analysis that included both randomized controlled trials (RCTs) and observational studies evaluating the benefit of upfront HDT/ASCT published during 2012 to 2023. Further sensitivity analysis and meta-regression were also performed.

**Results:**

Among the 22 enrolled studies, 7 RCTs and 9 observational studies had a low or moderate risk of bias, while the remaining 6 observational studies had a serious risk of bias. HDT/ASCT revealed advantages in complete response (CR) with an odds ratio (OR) of 1.24 and 95% confidence interval (CI) 1.02 ~ 1.51, PFS with a hazard ratio (HR) of 0.53 (95% CI 0.46 ~ 0.62), and OS with an HR of 0.58 (95% CI 0.50 ~ 0.69). Sensitivity analysis excluding the studies with serious risk of bias and trim-and-fill imputation fundamentally confirmed these findings. Older age, increased percentage of patients with International Staging System (ISS) stage III or high-risk genetic features, decreased proteasome inhibitor (PI) or combined PI/ immunomodulatory drugs (IMiD) utilization, and decreased follow-up duration or percentage of males were significantly related to a greater survival advantage with HDT/ASCT.

**Conclusions:**

Upfront ASCT remains a beneficial treatment for newly diagnosed MM patients in the period of novel agents. Its advantage is especially acute in high-risk MM populations, such as elderly individuals, males, those with ISS stage III or high-risk genetic features, but is attenuated with PI or combined PI/IMiD utilization, contributing to divergent survival outcomes.

**Supplementary Information:**

The online version contains supplementary material available at 10.1186/s12885-023-10907-1.

## Introduction

Multiple myeloma (MM), one of the most common hematologic malignancies, is characterized by abnormal monoclonal expansion of plasma cells in the bone marrow [1]. MM accounted for 155,688 newly diagnosed cases in 2019 and approximately 100,000 deaths annually worldwide [2]. Upfront high-dose therapy (HDT) with melphalan followed by autologous stem cell transplantation (ASCT) has proven to be effective and has become the standard treatment for eligible newly diagnosed MM patients in the past 30 years [3, 4]. ASCT allows a rapid restoration of bone marrow function after HDT [1]. Consequently, induction therapy, subsequent HDT/ASCT, and optional subsequent consolidation and maintenance therapy comprise the current fundamental framework for MM care [3].

In addition to traditional chemotherapeutic agents such as melphalan, doxorubicin, and cyclophosphamide, novel drugs are continuously emerging in this era, including proteasome inhibitors (PIs) (such as bortezomib, carfilzomib, and ixazomib), immunomodulatory drugs (IMiDs) (such as thalidomide, lenalidomide, and pomalidomide), anti-CD38 monoclonal antibodies (daratumumab), signaling lymphocytic activation molecule family member 7 (SLAMF7) inhibitors (elotuzumab), and histone deacetylase (HDAC) inhibitors (panobinostat) [5]. The incorporation of these agents into the treatment regimens substantially improves the survival outcome of MM patients, which ironically challenges the rationale of upfront HDT/ASCT [4, 6].

To address the current issue about the necessity of upfront HDT/ASCT for MM, in 2018 and 2019, Dhakal et al. [7] and Su et al. [8] performed 2 meta-analyses, both demonstrating that HDT/ASCT remains a beneficial treatment approach for newly diagnosed MM patients in the period of novel agents. However, in these 2 studies, although HDT/ASCT was related to significantly better progression-free survival (PFS) than standard-dose therapy (SDT) without ASCT, there was a nonsignificant difference in overall survival (OS) between the 2 groups. These 2 meta-analyses included 4 identical randomized controlled trials (RCTs) and disregarded observational studies in the era of novel agents. The limited number of included articles in these meta-analyses makes it difficult to explain the discrepancy between PFS and OS. In addition, studies with more potent induction regimens, including carfilzomib- or pomalidomide-based therapies, were not included. Therefore, an extended scale meta-analysis consisting of both RCTs and observational studies may provide complementary information about the benefit of upfront HDT/ASCT. In this systematic review and meta-analysis, a total of 22 studies from the past 10 years were utilized to clarify the roles of upfront HDT/ASCT for MM patients. Further meta-regression was also performed to explain the heterogeneity among the included studies.

## Methods

### Data sources and study selection

This study was conducted in compliance with the Preferred Reporting Items for Systematic Reviews and Meta-analyses (PRISMA) guidelines [9]. Using the search terms “ASCT”, “MM”, “publication year 2012–2022” (which was extended to 2023/03 in revision), “randomized controlled trial”, “comparative study”, and “observational study” in the Embase, Cochrane Library, and PubMed (MEDLINE) databases, potentially eligible studies that met the following criteria were included: (1) subjects were newly diagnosed MM patients; (2) the study compared ASCT with no ASCT; (3) the study reported the post-ASCT/consolidation complete response (CR) rate, PFS or OS; and (4) the publication year was after 2012. The exclusion criteria were as follows: (1) review articles; (2) conference presentations; (3) subjects were refractory MM patients who received salvage ASCT or not; and (4) the study compared early vs. delayed ASCT.

### Risk of bias assessment

The revised Cochrane risk-of-bias tool for randomized trials (RoB2) [10] and the Cochrane risk of bias tool for nonrandomized studies (ROBINS-I) [11] were adopted to assess the risk of bias in the RCTs and observational studies, respectively. The summary plots were generated using the robvis tool (https://mcguinlu.shinyapps.io/robvis/) [12].

### Data extraction

Four reviewers (CML, LCC, CYY, and FMT) independently reviewed the articles and abstracted the data. The following data were extracted from each selected article: study design, first author, publication year, number of patients, median or mean age of patients, proportion of patients with International Staging System (ISS) stage III classification, proportion of patients with high-risk cytogenetic features such as 1p deletion, 17p deletion, t(4;14) translocation, t(14;16) translocation, t(14;20) translocation, and 1q gain [13], median follow-up period, proportion of male patients, enrollment periods, treatment regimens, and outcomes including CR, PFS, and OS. If the article merely provided the ages in each subgroup, the median or mean age of the 2 arms was summarized and divided by 2 to denote the age of the overall population. The percentages of PI and IMiD utilization were either documented according to the exact records or estimated by equal division of the kinds of regimens if the real number was not available.

CR encompassed stringent complete response/remission (sCR), which is a more in-depth status of CR, while very good partial response (VGPR) was not counted. Only the post-ASCT/consolidation CR rate accounted for the overall CR rate, while the post-induction CR rate before ASCT/consolidation was excluded from the meta-analysis. For PFS and OS, the between-arm hazard ratio (HR) was extracted from each study if available. For those studies that did not report the HR directly, the HR was evaluated from the Kaplan‒Meier curve and the at-risk table. The value of survival probability in the curve was identified using WebPlotDigitizer software (version 4.5) (https://automeris.io/WebPlotDigitizer/), and the estimated HR with its 95% confidence interval (CI) was then measured using a HR calculation spreadsheet created by Tierney et al. [14].

### Statistical analysis

Statistical analysis was performed using Review Manager (RevMan) version 5.4 (The Cochrane Collaboration, Oxford, England) and Stata version 16.0 (College Station, Texas). Odds ratios (ORs) for the CR rate and HRs for PFS and OS were used to quantify the effect size. Based on a random effects model, the Mantel‒Haenszel (M-H) and inverse variance (IV) methods were used to analyze the OR and HR, respectively. The combined OR, HR, and their 95% CIs were illustrated by forest plots with 2 subgroups: RCTs and observational studies. Publication bias was assessed by the funnel plot in RevMan and the Egger’s test in Stata based on a random effects model and the restricted maximum likelihood (REML) method. The trim-and-fill method for adjusting for publication bias and the corresponding contour-enhanced funnel plot were implemented in Stata based on a random effects DerSimonian‒Laird method, which is the mode most comparable with the random effects M-H and IV methods in RevMan. In the contour-enhanced funnel plot, the imputed study located in the area of *P* > 0.1 represents a real adjustment for publication bias. Otherwise, the imputed study may adjust the plot asymmetry caused by other problems, such as heterogeneity. Heterogeneity between studies was evaluated by Cochran’s Q test and quantified with the *I*^*2*^ statistic. An *I*^*2*^ less than 25%, between 26–74%, and more than 75% denoted low, moderate, and high heterogeneity, respectively[15]. Univariate meta-regression and bubble plots were further used to depict the factors that possibly accounted for the heterogeneity.

## Results

### Characteristics of the studies included in the meta-analysis

**Fig. 1:**
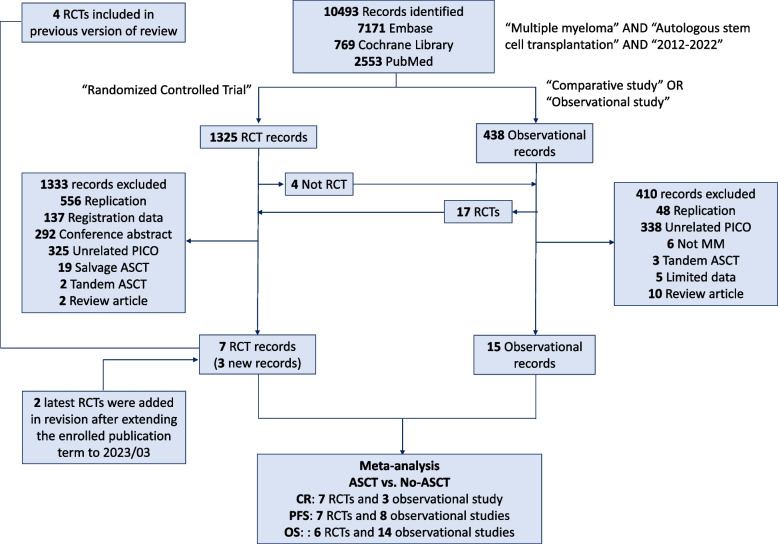
Study designs for this meta-analysis. RCT, randomized controlled trial; ASCT, autologous stem-cell transplantation; PICO, patient, intervention, comparison, and outcome; CR, complete response; PFS, progression-free survival; OS, overall survival

A total of 10,493 articles were identified initially, including 1325 RCTs and 438 observational studies. After excluding the studies that did not meet the inclusion criteria, 5 RCTs and 15 observational studies were included in the meta-analysis to compare the treatment outcomes between upfront ASCT and no upfront ASCT (briefly no ASCT) [16–35]. Two latest RCTs were added into the meta-analysis in the revised version after extending the enrolled publication term to 2023/03 [36, 37]. Figure 1 and Table 1 showed the research design and the baseline characteristics of the included studies. Among the 7 RCTs, 4 RCTs published during 2014 ~ 2020 were included in the previous versions of the review, whereas 3 RCTs published during 2021 ~ 2023 were newly enrolled [7, 8]. One of the 3 new RCTs (Gay et al., 2021) had a 3-arm design. In this RCT, 2 of the 3 arms (carfilzomib, lenalidomide, dexamethasone [KRD] + ASCT vs. KRD) were extracted for meta-analysis, while the KCD + ASCT arm (C: cyclophosphamide) was excluded due to a lack of comparative KCD alone group (Supplementary Table S1). The latest PI carfilzomib was used in 4 studies [24, 20, 34, 37], and the latest IMiD pomalidomide was mentioned in 1 study [25]. These 2 regimens were not included in the previous reviews [7, 8].

**Table 1:** Characteristics of the studies included in the meta-analysis

Studies	Number of patients (ASCT/No-ASCT)	Median or mean age (years)	ISS III (%)	High-risk genetics (%)	Median follow-up (months)	Male (%)	Enrollment periods ≥ 2012 (%)	Induction (PI %) (IMiD %) (PI + IMiD %) (Triplet %)	Consolidation (ASCT/No-ASCT)	Maintenance	Outcomes
RCTs											
Palumbo et al., 2014 [16]	273 (141/132)	57	23.6	28.8	51.2	59	2007 ~ 2009 (0)	RD (0) (100) (0) (0)	M200/MPR	R	CR, PFS, OS
Gay et al., 2015 [17]	256 (127/129)	56.5	29	21.8	52	46.9	2009 ~ 2011 (0)	RD (0) (100) (0) (0)	M200/CRD	R	CR, PFS, OS
Attal et al., 2017 [18]	700 (350/350)	59.5	18	12.8	44	60.3	2010 ~ 2012 (33.3)	RVD (100) (100) (100) (100)	M200/RVD	R	CR, PFS, OS
Cavo et al., 2020 [19]	1197 (702/495)	58	20.1	25	60.3	57.7	2011 ~ 2014 (75)	CVD (100) (0) (0) (100)	(M200/VMP) → RVD (37.5%)	R	CR, PFS, OS
Gay et al., 2021 [20]	315 (158/157)	57	17.1	47	50.9	55.6	2015 ~ 2017 (100)	KRD (100) (100) (100) (100)	M200/KRD	KR or R	CR, PFS, OS
Richardson et al., 2022 [36]	722 (365/357)	56	13.3	18.3	76	57.8	2010 ~ 2018 (77.8)	RVD (100) (100) (100) (100)	M200/RVD	R	CR, PFS, OS
Yong et al., 2023 [37]	218(109/109)	59	19	20	40.2	59	After 2015 (100)	KCD (100) (0) (0) (100)	M (high dose)/KCD	K	CR, PFS
Observational studies											
Wildes et al., 2015 [21]	146 (62/84)	68	NA	NA	48.4	52.7	2000 ~ 2010 (0)	T, R, V, or others (24.9^e^) (49.9^e^) (NA) (NA)	M200/NA	None, T, R, V, or others	OS
Biran et al., 2016 [22]	431 (90/341)	62	23.4	4.9	NA	55.9	2004 ~ 2006 (0)	RD (0) (100) (0) (0)	NA	NA	OS
Cohen et al., 2018 [23]	60 (34/26)	62	35	100	17	20	2008 ~ 2016 (55.6)	V, CVD, or others (88) (NA) (NA) (≥ 72)	NA	NA	PFS, OS
Hajek et al., 2018 [24]	2446 (710/1736) for OS 2442 (709/1733) for PFS 977 (236/741) for CR	67	35.8	NA	22.2	53.3	2007 ~ 2014 (37.5)	V, K, R, T, or others (48.2) (45.3) (4.9) (NA)	NA	NA	CR, PFS, OS
Remes et al., 2018 [25]	275 (114/161)	66	NA	NA	25	43	2009 ~ 2013 (40)	R, T, V, or Pom (25^e^) (75^e^) (NA) (NA)	NA	NA	TTNT (PFS), OS
Rosenberg et al., 2019 [26]	5309 (2125/3184)	61.5	NA	NA	98.5	55	1998 ~ 2012 (6.7)	N/A	NA	NA	OS
Belotti et al., 2020 [27]	131 (85/46)	70.7	34	10	27	50.4	2013 ~ 2017 (100)	V(T)D, CVD, or others (97.7) (61.1) (61.1) (94.7)	M200/NA	NA	CR, PFS
Czyż et al., 2020 [28]	97 (29/68)	63	35	100	33	56.7	2011 ~ 2017 (85.7)	(V)CTD or MTD (49) (61.9) (19.6) (100) (NA)	NA	NA	OS
Goldman-Mazur et al., 2020 [29]	230 (82/148)	62	48.4	NA	NA	40.2	2005 ~ 2018 (50)	PI, IMiD, or others (80.5) (51.8) (37.5) (NA)	NA	NA	PFS, OS
Kaur et al., 2021 [30]	939 (378/561)	65.6	15.5	20.5	NA	48.9	2000 ~ 2017 (33.3)	CVD or others (66^e^) (66^e^) (39.7) (≥ 39.7)	NA	NA	OS
Lemieux et al., 2021 [31]	79 (38/41)	71	26.6	89.9	40	64.6	2010 ~ 2019 (80)	PI, IMiD, or others (88.6) (64.6) (59.5) (82.3)	NA	NA	PFS, OS
Abello et al., 2022 [32]	872 (252/620)	67	35.1	NA	18	52.9	2018 ~ 2020 (100)	CVD, VTD, or others (79.2) (30.8) (24) (74.4)	NA	NA	OS
Bai et al., 2022 [33]	42 (25/17)	69	47.6	NA	NA	57.1	2010 ~ 2018 (77.8)	VTD (100) (100) (100) (100)	M200/NA	NA	CR, OS
Cho et al., 2022 [34]	210 (54/156)	67	19.5	9	34.5	51.4	2011 ~ 2018 (87.5)	VMP, KMP, VTD, or CVD (80) (45.7) (25.7) (≥ 80)	NA	NA	PFS, OS
Pawlyn et al., 2022 [35]	770 (404/366)	67	25.3	44.1	NA	61.2	2013 ~ 2017 (100)	CTD or CRD (0) (100) (0) (100)	NA	R ± Vor	PFS, OS

*RCT* Randomized controlled trial, *ASCT* Autologous stem-cell transplantation, *ISS* International staging system, *R* lenalidomide, *V* Bortezomib, *D* Dexamethasone, *C* Cyclophosphamide, *K* Carfilzomib, *T* Thalidomide, *M* Melphalan 100 mg/m^2^, *M200* Melphalan 200 mg/m^2^, *P* Prednisone, *Pom* Pomalidomide, *Vor* Vorinostat, *PI* Protease inhibitor, *IMiD* Immunomodulatory agent, *CR* Complete response, *PFS* Progression-free survival, *TTNT* Time to next treatment, *OS* Overall survival, *NA* Not applicable, *e* Estimated by equal division of the kinds of the regimens

### Risk of bias assessment of the studies included in the meta-analysis

**Fig. 2:**
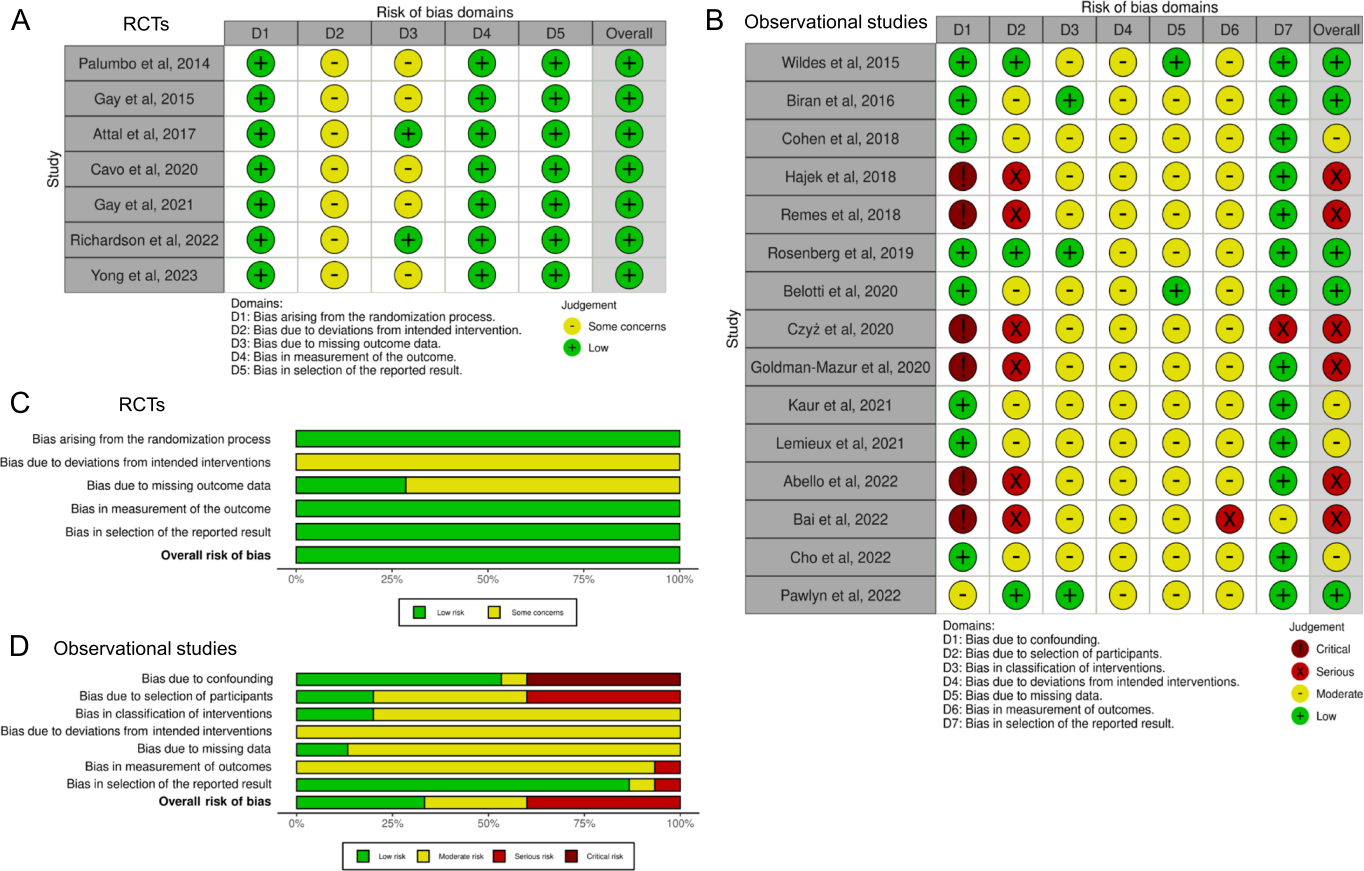
Risk-of-bias assessment. **A**, **C**. The revised Cochrane risk-of-bias tool for randomized trials (RoB2) of the RCT studies included in the meta-analysis; **B**, **D**. The Cochrane risk of bias tool for non-randomized studies (ROBINS-I) of the observational studies in the meta-analysis. RCT, randomized controlled trial

As seen in Supplementary Table S1, the RCTs were all open-label and multicenter, with a similar randomization process stratified by age, ISS, or other factors. The drop-out rates of these RCTs after randomization were predominantly higher in the no ASCT group than in the ASCT group, contributing to a possible attrition bias. In the RoB2 assessment (Fig. 2A and C), there were some concerns of bias due to deviations from the intended intervention in all 7 RCTs because of the inevitable open-label design. Five RCTs with obvious distinct drop-out rates between the ASCT and no ASCT groups raised some concerns of bias due to missing outcome data. Overall, the risk of bias in all 7 RCTs remained low. In contrast, the risk of bias in 6 observational studies was serious because they did not utilize common methodologies for bias control (Supplementary Table S2, Fig. 2B and D). Four observational studies were considered to have a moderate risk of bias because they utilized only one methodology to control for bias. The other 5 observational studies were regarded to have a low risk of bias because they executed a prospective intention-to-treat (ITT) cohort design or adopted multiple methods to control for bias and confounders. Regarding the study by Lemieux et al., multivariate regression adjustment was only applied for PFS, not for OS, increasing the bias risk of OS from moderate to serious. The PFS advantage of ASCT compared to no ASCT was strengthened after adjustment in the study by Lemieux et al., while the PFS and OS benefits of ASCT were attenuated after adjustment in the studies by Belotti et al. and Biran et al., respectively (Supplementary Table S3). For those observational studies that provided the adjusted HRs, the adjusted HRs were used for pooling outcomes. Otherwise, the unadjusted HRs and the HRs estimated from the Kaplan‒Meier curves were adopted for the following meta-analysis. Those unadjusted/estimated HRs were excluded from the sensitivity analysis to verify the robustness of the major outcomes.

### Meta-analysis comparing the treatment outcomes between ASCT and no ASCT

**Fig. 3:**
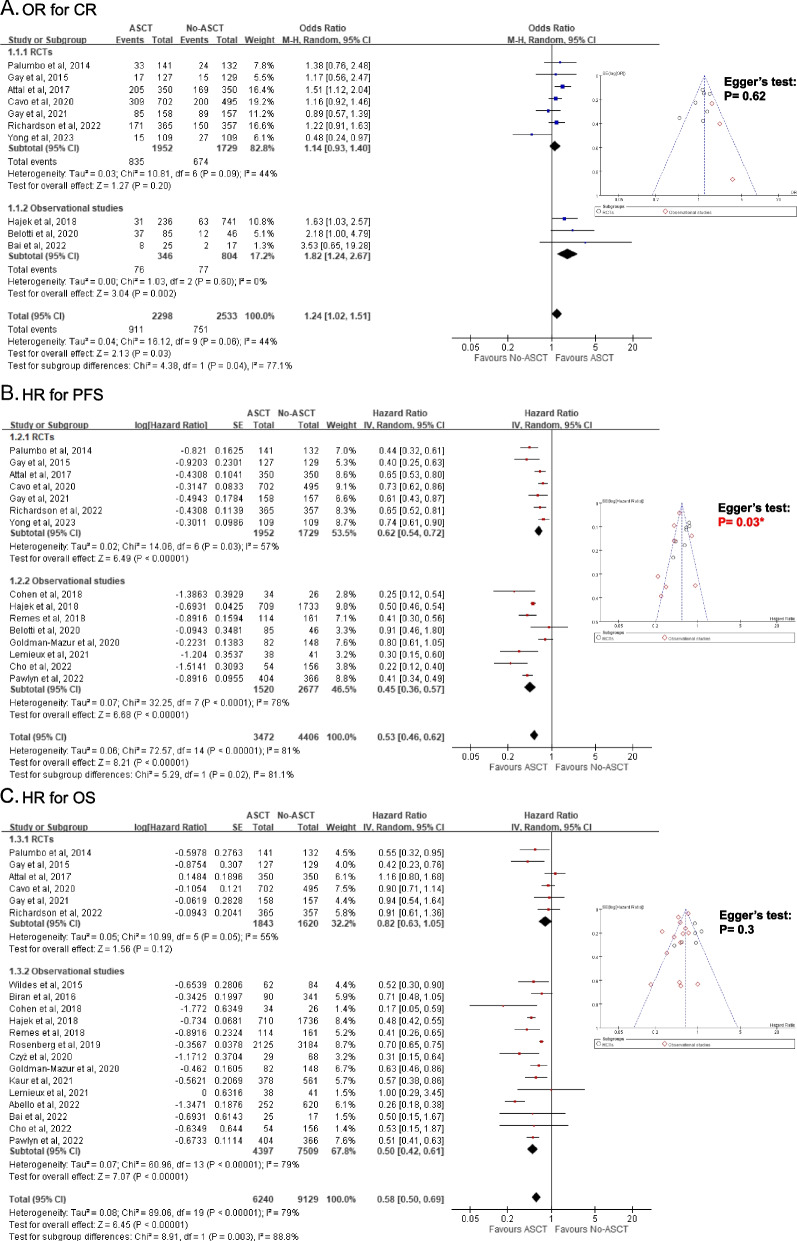
Forest and funnel plots of the meta-analysis comparing ASCT and No-ASCT. A. OR of CR; B. HR of mortality for PFS; C: HR of mortality for OS. RCT, randomized controlled trial; ASCT, autologous stem-cell transplantation; CR, complete response; PFS, progression-free survival; OS, overall survival; OR, odds ratio; HR, hazard ratio; M-H, Mantel–Haenszel method; IV, Inverse variance; SE, standard error; CI, confidence interval; *statistical significance

As shown in Supplementary Table S3, the survival outcomes of 5 observational studies were transformed into HRs and added to the meta-analysis. Regarding CR, the M-H random method reported a significantly higher CR rate in the ASCT group (OR 1.24, 95% CI 1.02 ~ 1.51) (Fig. 3A). An *I*^*2*^ of 44% indicated a moderate probability of heterogeneity, which was more likely to be attributed to the RCT by Yong et al. with a contrasting tendency. This non-inferiority trial only randomized the patients with at least partial responses to the induction chemotherapy, which may be profitable to the no ASCT group. The nonsignificant Egger’s test implied a low probability of publication bias. Though the sensitivity analysis that excluded 2 studies with a high risk of bias insignificantly favored ASCT, the other 2 kinds of sensitivity analyses that excluded the RCT by Yong et al. significantly favored ASCT over no ASCT for the CR rate (Supplementary Fig. S1A-C). The trim-and-fill method imputed 1 more study and still demonstrated a preference for ASCT for the CR rate (Supplementary Table S5 and Supplementary Fig. S2A). Regarding PFS, the IV random method depicted a significantly better PFS in the ASCT group (HR 0.53, 95% CI 0.46 ~ 0.62) (Fig. 3B). An *I*^*2*^ of 81% and a significant Egger’s test (*P* = 0.03) implied a high possibility of heterogeneity and publication bias. Similar results could be seen in the sensitivity analysis, from which 3 studies with a high risk of bias were excluded (Supplementary Fig. S1D). The significant benefit of ASCT for PFS was sustained even after trim-and-fill imputation for publication bias adjustment (Supplementary Table S5 and Supplementary Fig. S2B). Regarding OS, although an overall significantly better prognosis was observed in the ASCT group (HR 0.58, 95% CI 0.50 ~ 0.69), there was no significant benefit for the ASCT group in the RCTs alone (Fig. 3C). An *I*^*2*^ of 79% and a nonsignificant Egger’s test delineated a high level of heterogeneity but a low risk of publication bias. The sensitivity analysis that excluded 7 studies with a high risk of bias revealed a comparable result (Supplementary Fig. S1E). The trim-and-fill method also demonstrated a sustained advantage of ASCT for OS after imputation (Supplementary Table S5 and Supplementary Fig. S2C).

### Meta-regression for PFS and OS when comparing ASCT with no ASCT

**Fig. 4:**
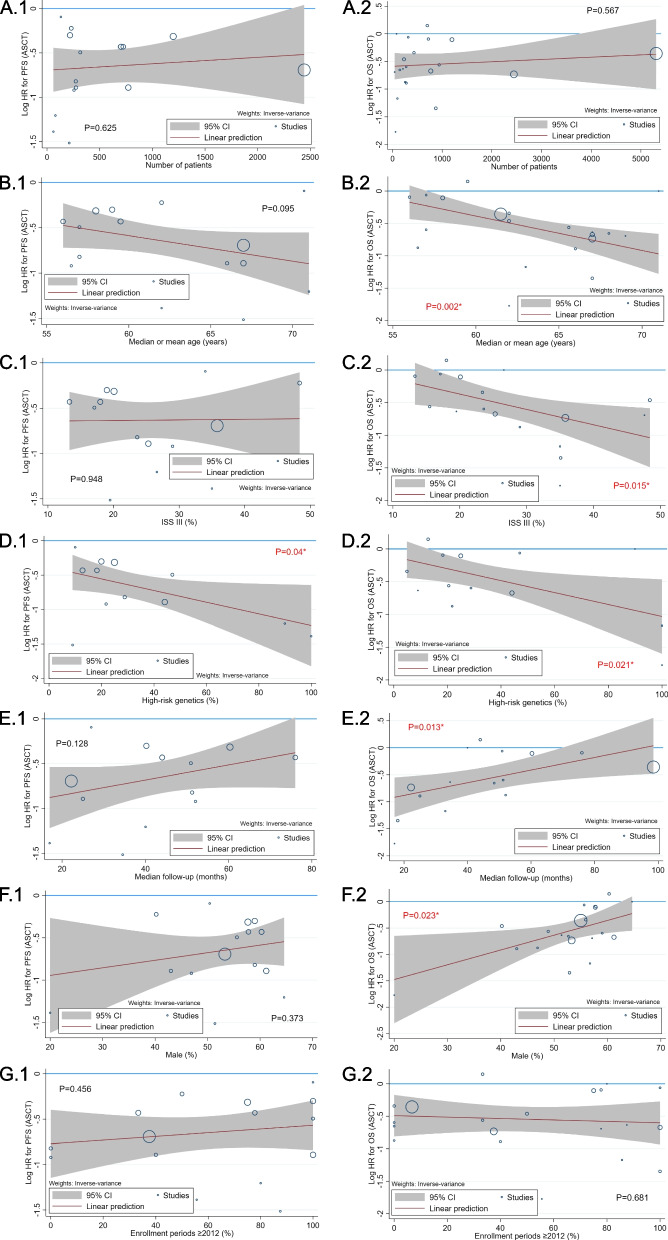
Bubble plots of meta-regression regarding the HR of ASCT compared with No-ASCT for PFS and OS, according to the characteristics of the enrolled patients. 1: PFS; 2: OS; **A**: number of patients; **B**: median or mean age; **C**: ISS III; **D**: high-risk genetics; **E**: median follow-up; **F**: male; **G**: enrollment periods ≥ 2012; ASCT, autologous stem-cell transplantation; ISS, international Staging System; HR, hazard ratio; PFS, progression-free survival; OS, overall survival; * statistical significance

**Fig. 5:**
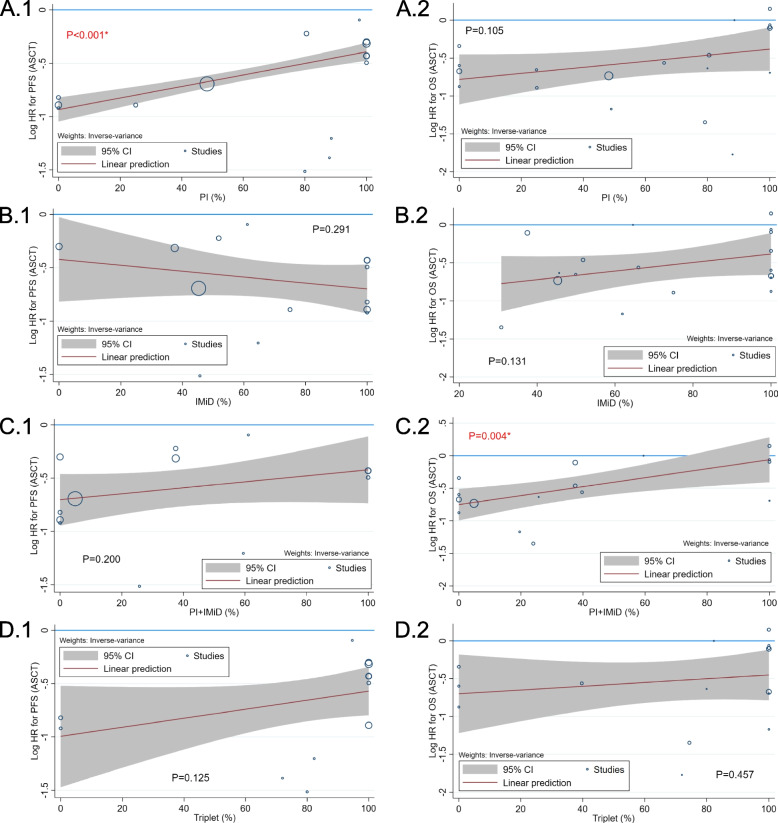
Bubble plots of meta-regression regarding the HR of ASCT compared with No-ASCT for PFS and OS, according to the induction/consolidation regimens. 1: PFS; 2: OS; A: PI; B: IMiD; C: Combined PI and IMiD; D: Triplet regimen; ASCT, autologous stem-cell transplantation; PI, protease inhibitor; IMiD, immunomodulatory agent; HR, hazard ratio; PFS, progression-free survival; OS, overall survival; * statistical significance

Seven possible characteristics of the enrolled patients contributing to the heterogeneity were identified after data analysis, including the number of patients, median or mean age, percentage of patients with ISS stage III, percentage of patients with high-risk genetic features, median follow-up duration, percentage of males, and percentage of enrolled periods after 2012 (Table 1). As shown in Fig. 4, meta-regression revealed that increased high-risk genetic features were significantly related to a better PFS and OS benefit of ASCT. Older median or mean age, increased percentage of patients with ISS stage III, decreased median follow-up duration, and decreased percentage of males were significantly associated with an improved OS with ASCT. Regarding the treatment regimens of PI, IMiD, combined PI/IMiD (at induction and consolidation phase), and triplet (a 3 or more combination of traditional agents/novel agents/steroids at induction phase), the meta-regression demonstrated that increased percentage of PI and combined PI/IMiD utilization was significantly associated with a lower PFS and OS benefit of ASCT, respectively (Fig. 5).

## Discussion

In this meta-analysis, we included both RCTs and observational studies published during 2012 ~ 2023 to estimate the effect size of upfront HDT/ASCT in MM treatment, involving the usage of the latest PI carfilzomib and IMiD pomalidomide. Among the 22 included articles, all 7 RCTs were considered to have a low risk of bias, while 6 observational studies lacked methodologies to control for potential bias and were regarded to have a serious risk of bias. Upfront HDT/ASCT was related to a significantly better CR, PFS, and OS than SDT/no ASCT. Two kinds of sensitivity analyses, including the removal of the studies with a high risk of bias and the trim-and-fill imputation method, fundamentally confirmed these findings. Finally, the meta-regression analysis revealed that decreased proportion of PI or combined PI/IMiD utilization, decreased percentage of males or median follow-up duration, older median or mean age, and increased percentage of patients with ISS stage III or high-risk genetic features were significantly associated with an improved PFS or OS for upfront HDT/ASCT in MM care.

Previous meta-analyses in 2018 and 2019 revealed significant PFS benefits but nonsignificant CR and OS advantages with HDT/ASCT in treatment for newly diagnosed MM patients [7, 8]. One possibility to explain this disparity is that salvage HDT/ASCT is frequently employed upon relapse for the SDT/no ASCT group, which diminishes the OS benefits witnessed in the upfront HDT/ASCT group [7]. Another possibility may be related to the low statistical power due to the limited numbers of enrolled studies (4 RCTs in previous meta-analyses) to detect the real effects of HDT/ASCT, which can be reinforced by increasing the numbers of recruited articles. By adding 3 extra RCT and 15 observational studies to raise the statistical power, our meta-analysis revealed a uniform advantage of HDT/ASCT in CR, PFS, and OS compared to SDT/no ASCT for MM treatment. Generally, observational studies have a higher risk of bias than RCTs. Therefore, including observational studies in this meta-analysis inevitably reduced its level of evidence. However, through rigorous risk of bias assessments, adjusted outcome utilization, and supplementary sensitivity analyses that constricted or imputed the included studies in our meta-analysis, we could exploit these observational studies to enrich previous findings derived from the RCTs.

Our meta-regression demonstrated that age, sex, high-risk disease status, and follow-up duration may influence the OS benefit of HDT/ASCT, which is likely to be another explanation accounting for the discrepant results between PFS and OS in the previous meta-analyses. The longer the follow-up duration is, the higher the possibility that salvage HDT/ASCT and the newest potent treatment regimens will be applied in the SDT/no ASCT group, which might reduce the OS difference between the 2 groups. It has been reported that older age is a crucial risk factor for elevated MM mortality, possibly due to deterioration of the general condition and intolerance to high-intensity antitumor therapy [38, 39]. Previous studies also implied that males have higher incidence and mortality rates than females, which might be attributed to hormone differences and an increased probability of carcinogen exposure among males, such as smoking and alcohol [38, 40]. HDT/ASCT was reported to be effective for MM patients with both high- and low-risk genetic features [41]. Altogether, our findings indicate that SDT/no ASCT may have fewer effects on high-risk MM patients (including elderly individuals, males, those with ISS stage III, and those with high-risk genetic features) than HDT/ASCT.

In our study, although the enrolled periods and IMiD utilization did not have a significant influence on the PFS and OS advantage of HDT/ASCT, increased PI and combined PI/IMiD usage significantly attenuated the PFS and OS benefit of HDT/ASCT. This finding is consistent with previous research in which the PFS and OS benefits of HDT/ASCT seemed to be less marked in the group treated with PI bortezomib-based regimen than in the group treated with traditional alkylating agent-based regimen [8]. This implies that though the current novel agents are not strong enough to replace ASCT in MM treatment, their usage has brought noticeable survival advantages, thus diminishing the benefits of ASCT. More potent regimens that emerge in the future will keep challenging the rationale of HDT/ASCT in MM care.

Some limitations in our meta-analysis must be noted. First, the inclusion of observational studies may increase the risk of bias and attenuate the strength of the evidence in this article. Second, the HRs in 5 observational studies were estimated from the Kaplan‒Meier curves, which could increase the risk of measurement bias. Third, most of the observational studies lacked data on maintenance regimens, making it difficult to analyze the impact of such regimens on survival. Forth, the percentages of PI and IMiD usage in 3 articles were estimated by equal division of the kinds of regimens, which was not in accordance with the actual distribution. Fifth, we excluded those studies that compared tandem vs. single ASCT and early vs. delayed ASCT; the times and timing of upfront ASCT are also likely to alter the HDT/ASCT effects in MM treatment.

To the best of our knowledge, this is the first meta-analysis to combine both RCTs and observational studies to compare ASCT with no ASCT and to identify possible factors that influence ASCT advantages for newly diagnosed MM patients in the era of novel drugs. Our analysis revealed that upfront ASCT has CR, PFS, and OS benefits. Older age, increased percentage of people with ISS stage III or high-risk genetic features, decreased PI or combined PI/IMiD utilization, and decreased follow-up duration or percentage of males are related to a greater survival advantage with ASCT, which probably accounts for the discrepancy between PFS and OS in the previous meta-analyses. In brief, upfront ASCT remains a beneficial treatment for newly diagnosed MM patients in the era of novel agents. Its advantage is particularly pronounced in high-risk MM populations, such as elderly individuals, males, those with ISS stage III, and those with high-risk genetic features; however, it is attenuated with PI or combined PI/IMiD utilization, leading to divergent survival outcomes.

## Supplementary Information

**Additional file 1: Supplementary Table S1.** Qualities of the RCT studies. **Supplementary Table S2.** Qualities of the observational studies. **Supplementary Table S3.** Main results of the observational studies before and after adjustment. **Supplementary Table S4.** Main results of the observational studies before and after transformation. **Supplementary Table S5.** Trim and fill method for adjustment of publication bias. **Supplementary Figure S1.** Forest plots of the meta-analysis comparing ASCT and No-ASCT, excluding the studies with a serious risk of bias or a specialized design. A. OR of CR (analysis 1) excluding the studies with a serious risk of bias; B. OR of CR (analysis 2) excluding the studies with a specialized design; C. OR of CR (analysis 3) excluding the studies with a serious risk of bias and a specialized design; D. HR of mortality for PFS; E: HR of mortality for OS. RCT, randomized controlled trial; ASCT, autologous stem-cell transplantation; CR, complete response; PFS, progression-free survival; OS, overall survival; OR, odds ratio; HR, hazard ratio; M-H, Mantel Haenszel method; IV, Inverse variance; SE, standard error; CI, confidence interval. **Supplementary Figure S2. **Contour funnel plots of trim and fill method. A: OR for CR; B: HR for PFS; C: HR for OS; CR, complete response; OR. odds ratio; HR, hazard ratio; PFS, progression-free survival; OS, overall survival; SE, standard error.

## Data Availability

The data generated in this study are available within the article and its supplementary data files.
