# Supplementary material for: Treatment benefit of upfront autologous stem cell transplantation for newly diagnosed multiple myeloma: a systematic review and meta-analysis
Source: BMC Cancer. 2023 May 16;23:446. doi: 10.1186/s12885-023-10907-1 (PMC10190007; doi:10.1186/s12885-023-10907-1)
Supplement: Supplementary file 1 — Additional file 1: Supplementary Table S1. Qualities of the RCT studies. Supplementary Table S2. Qualities of the observational studies. Supplementary Table S3. Main results of the observational studies before and after adjustment. Supplementary Table S4. Main results of the observational studies before and after transformation. Supplementary Table S5. Trim and fill method for adjustment of publication bias. Supplementary Figure S1. Forest plots of the meta-analysis comparing ASCT and No-ASCT, excluding the studies with a serious risk of bias or a specialized design. A. OR of CR (analysis 1) excluding the studies with a serious risk of bias; B. OR of CR (analysis 2) excluding the studies with a specialized design; C. OR of CR (analysis 3) excluding the studies with a serious risk of bias and a specialized design; D. HR of mortality for PFS; E: HR of mortality for OS. RCT, randomized controlled trial; ASCT, autologous stem-cell transplantation; CR, complete response; PFS, progression-free survival; OS, overall survival; OR, odds ratio; HR, hazard ratio; M-H, Mantel Haenszel method; IV, Inverse variance; SE, standard error; CI, confidence interval. Supplementary Figure S2. Contour funnel plots of trim and fill method. A: OR for CR; B: HR for PFS; C: HR for OS; CR, complete response; OR. odds ratio; HR, hazard ratio; PFS, progression-free survival; OS, overall survival; SE, standard error. [file 12885_2023_10907_MOESM1_ESM.pdf]

Supplementary Table S1. Qualities of the RCT studies

Studies	Multicenter	Randomization	Drop Out After Randomization	Power (Predicted difference)	Estimated/Actual overall sample size
Palumbo et al, 2014	Yes	Stratified by age, ISS	ASCT, 4.2%; No-ASCT, 12.1%	85% for 2-year PFS (ASCT HR 0.62)	400/402 (129 discontinued before randomization)
Gay et al, 2015	Yes	Stratified by age, ISS	ASCT, 7.9%; No-ASCT, 17.8%	80% for 2-year PFS (ASCT HR 0.7)	390/389 (133 discontinued before randomization)
Attal et al, 2017	Yes	Stratified by ISS, FISH	ASCT, 5%; No-ASCT, 8%	80% for ≥ 9-months PFS (ASCT 39, No-ASCT 30 months)	700/700
Cavo et al, 2020	Yes	Stratified by site, ISS	ASCT, 19%; No-ASCT, 37.6%	80% for PFS (ASCT HR 0.78)	1202/1354 (157 discontinued before randomization)
Gay et al, 2021	Yes	Stratified by age, ISS	ASCT, 19%; No-ASCT, 28%	90% for ≥VGPR (KRD: 80%; KCD:62%)	477/477 (3 arms: KRD+ASCT, KRD, and KCD+ASCT)
Richardson et al, 2022	Yes	Stratified by ISS, FISH	ASCT, 20.8%; No-ASCT, 18.5%	90% for PFS (ASCT HR 0.7)	722/720 (151 discontinued before randomization)
Yong et al, 2023	Yes	Stratified by hospital, response to induction, ISS, FISH	ASCT, 4.6%; No-ASCT, 9.2%	80% for 2-year PFS (10% non-inferiority margin)	210/218 (63 discontinued before randomization)

RCT, randomized controlled trial; ASCT, autologous stem-cell transplantation; FISH, fluorescence in situ hybridization; ISS, international Staging System; RVD, lenalidomide, bortezomib, and dexamethasone; KRD, carfilzomib, lenalidomide, and dexamethasone; KCD, carfilzomib, cyclophosphamide, and dexamethasone; PFS, progression-free survival; VGPR, very good partial response; HR, hazard ratio.

Supplementary Table S2. Qualities of the observational studies

Studies	Design	Multicenter	Propensity score	Multivariate Regression	Immortal time bias adjustment	Missing values imputation
Wildes et al, 2015	Retrospective	No	Yes	Yes	Nil	Yes
Biran et al, 2016	Retrospective	Yes	Nil	Yes	Yes	Nil
Cohen et al, 2018	Retrospective	Yes	Nil	Yes	Nil	Nil
Hajek et al, 2018	Retrospective	Yes	Nil	Nil	Nil	Nil
Remes et al, 2018	Retrospective	Yes	Nil	Nil	Nil	Nil
Rosenberg et al, 2019	Retrospective	Yes	Yes	Yes	Yes	Nil
Belotti et al, 2020	Prospective; ITT	No	Nil	Yes	Nil	Nil
Czyż et al, 2020	Retrospective	Yes	Nil	Nil	Nil	Nil
Goldman-Mazur et al, 2020	Retrospective	Yes	Nil	Nil	Nil	Nil
Kaur et al, 2021	Retrospective	No	Nil	Yes	Nil	Nil
Lemieux et al, 2021	Retrospective	No	Nil	Yes (for PFS only)	Nil	Nil
Abello et al, 2022	Retrospective	Yes	Nil	Nil	Nil	Nil
Bai et al, 2022	Retrospective	No	Nil	Nil	Nil	Nil
Cho et al, 2022	Retrospective	Yes	Nil	Yes	Nil	Nil
Pawlyn et al, 2022	Retrospective	Yes	Yes	Nil	Yes	Nil

ITT, intention-to-treat.

Supplementary Table S3. Main results of the observational studies before and after adjustment

Studies	Outcome	Before adjustment	Adjustment	After adjustment
Wildes et al, 2015	OS	ASCT HR: 0.54 (0.35~0.82)	Propensity score; Performance status; Comorbidity; Durie salmon stage	ASCT HR: 0.52 (0.30~0.91)
Biran et al, 2016	OS	ASCT HR: 0.8 (0.72~0.88)	ASCT as time-varying covariate; Treatment; Age; ISS; ECOG	ASCT HR: 0.71 (0.48~1.04)
Cohen et al, 2018	PFS	ASCT HR: 0.43 (0.23~0.79)	M spike level; ISS; Hb; LDH; Extramedullary disease; % of cells with del 17	ASCT HR: 0.25 (0.056~0.54)
	OS	ASCT HR: 0.19 (0.07~0.51)	Age; M spike level; ISS; Hb; LDH	ASCT HR: 0.17 (0.02~0.59)
Rosenberg et al, 2019	OS	NA	ASCT as time-varying covariate; Propensity score	ASCT HR: 0.70 (0.65~0.75)
Belotti et al, 2020	PFS	ASCT HR: 0.42 (0.25~0.71)	Cytogenetics; ISS; IMWG frailty score	ASCT HR: 0.91 (0.46~1.80)
Kaur et al, 2021	OS	NA	Race; Age; IMWG risk classification; Sex; First-line response; Initial therapy	ASCT HR: 0.57 (0.38~0.87)
Lemieux et al, 2021	PFS	ASCT HR: 0.69 (0.42~1.13)	Treatment response; HCT-CI; Cytogenetics; Use of maintenance	ASCT HR: 0.30 (0.15~0.63)
Cho et al, 2022	PFS	NA	Age; ISS; Focal lesions	ASCT HR: 0.22 (0.12~0.43)
	OS	NA	ECOG; ISS; Focal lesions	ASCT HR: 0.53 (0.15~0.82)
Pawlyn et al, 2022	PFS	NA	Propensity score	ASCT HR: 0.41 (0.34~0.48)
	OS	NA	Propensity score	ASCT HR: 0.51 (0.41~0.64)

ASCT, autologous stem-cell transplantation; CR, complete response; PFS, progression-free survival; OS, overall survival; OR, odds ratio; HR, hazard ratio; (95% confidence interval); HCT-CI, hematopoietic cell transplantation comorbidity index; IMWG, International Myeloma Working Group; ISS, International Staging System; ECOG, Eastern Cooperative Oncology Group performance status

Supplementary Table S4. Main results of the observational studies before and after transformation

Studies	Outcome	Main results	Transformation
Hajek et al, 2018	PFS	Median (months) (ASCT vs. No-ASCT): 30.0 (27.7~32.3) vs. 11.6 (10.8~12.5). P<0.001	ASCT HR: 0.5 (0.46~0.55)
	OS	Median (months) (ASCT vs. No-ASCT): Not reached vs. 34.6 (31.6~37.5). P<0.001	ASCT HR: 0.48 (0.42~0.54)
Remes et al, 2018	PFS	Median (months) (ASCT vs. No-ASCT): 33.9 (27.8~) vs. 12.6 (10.2~15.8). P<0.001	ASCT HR: 0.41 (0.30~0.56)
	OS	Median (months) (ASCT vs. No-ASCT): Not reached vs. 46.2 (44~). P<0.001	ASCT HR: 0.41 (0.26~0.66)
Lemieux et al, 2021	PFS	Median (months) (ASCT vs. No-ASCT): 41 vs. 33. P=0.03	ASCT HR: 0.69 (0.42~1.13) (before adjustment)
	OS	5-year OS (ASCT vs. No-ASCT): 73% vs. 83%. P=0.86	ASCT HR: 1.00 (0.29~3.47)
Goldman-Mazur et al, 2020	PFS	No difference. P=0.097	ASCT HR: 0.8 (0.61~1.04)
	OS	Median (months) (ASCT vs. No-ASCT): 60.0 (47~101) vs. 37.9 (23.3~47). P<0.001	ASCT HR: 0.63 (0.46~0.87)
Bai et al, 2022	OS	1st, 2nd and 3rd year OS (%) (ASCT vs. No-ASCT): 96 vs. 70.59, 88 vs. 58.82, and 80 vs. 47.06. P<0.05	ASCT HR: 0.40 (0.13~1.23)

ASCT, autologous stem-cell transplantation; CR, complete response; PFS, progression-free survival; OS, overall survival; OR, odds ratio; HR, hazard ratio; (95% confidence interval)

Supplementary Table S5. Trim and fill method for adjustment of publication bias

ASCT	Egger's test	Original number of studies	Original effect size (95% CI)	Imputed number of studies	Imputed number of studies in the area of P>0.1	Total number of studies after imputation	Effect size after imputation (95% CI)
OR for CR	0.62	10	1.24 (1.02~1.51)	1	1	11	1.22 (1.001~1.49)
HR for PFS	0.03	15	0.53 (0.46~0.62)	2	2	17	0.56 (0.48~0.66)
HR for OS	0.3	20	0.58 (0.50~0.69)	1	1	21	0.60 (0.51~0.70)

ASCT, autologous stem-cell transplantation; CR, complete response; PFS, progression-free survival; OS, overall survival; OR, odds ratio; HR, hazard ratio; CI, confidence interval

A. OR for CR (analysis 1)

Study or Subgroup	ASCT Events	ASCT Total	No-ASCT Events	No-ASCT Total	Weight	Odds Ratio M-H, Random, 95% CI
**2.1.1 RCTs**						
Palumbo et al, 2014	33	141	24	132	8.8%	1.38 [0.76, 2.48]
Gay et al, 2015	17	127	15	129	6.2%	1.17 [0.56, 2.47]
Attal et al, 2017	205	350	169	350	18.7%	1.51 [1.12, 2.04]
Cavo et al, 2020	309	702	200	495	22.0%	1.16 [0.92, 1.46]
Gay et al, 2021	85	158	89	157	12.7%	0.89 [0.57, 1.39]
Richardson et al, 2022	171	365	150	357	18.9%	1.22 [0.91, 1.63]
Yong et al, 2023	15	109	27	109	6.9%	0.48 [0.24, 0.97]
**Subtotal (95% CI)**		**1952**		**1729**	**94.3%**	**1.14 [0.93, 1.40]**
Total events	835		674			
Heterogeneity: Tau² = 0.03; Chi² = 10.81, df = 6 (P = 0.09); I² = 44%						
Test for overall effect: Z = 1.27 (P = 0.20)						
**2.1.2 Observational study (excluding 2 studies)**						
Belotti et al, 2020	37	85	12	46	5.7%	2.18 [1.00, 4.79]
**Subtotal (95% CI)**		**85**		**46**	**5.7%**	**2.18 [1.00, 4.79]**
Total events	37		12			
Heterogeneity: Not applicable						
Test for overall effect: Z = 1.95 (P = 0.05)						
**Total (95% CI)**		**2037**		**1775**	**100.0%**	**1.18 [0.96, 1.45]**
Total events	872		686			
Heterogeneity: Tau² = 0.04; Chi² = 13.10, df = 7 (P = 0.07); I² = 47%						
Test for overall effect: Z = 1.56 (P = 0.12)						
Test for subgroup differences: Chi² = 2.45, df = 1 (P = 0.12), I² = 59.3%						

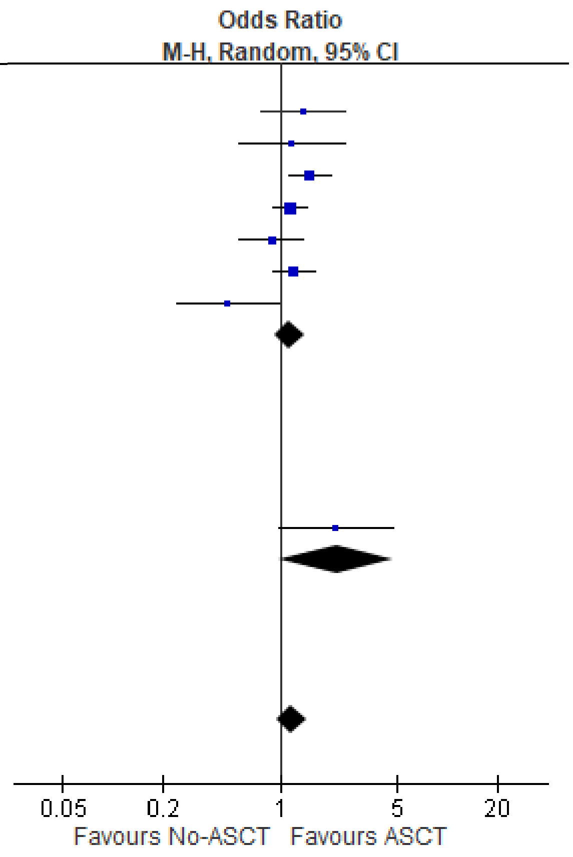

B. OR for CR (analysis 2)

Study or Subgroup	ASCT Events	ASCT Total	No-ASCT Events	No-ASCT Total	Weight	Odds Ratio M-H, Random, 95% CI
**2.2.1 RCTs (excluding 1 study)**						
Palumbo et al, 2014	33	141	24	132	5.7%	1.38 [0.76, 2.48]
Gay et al, 2015	17	127	15	129	3.7%	1.17 [0.56, 2.47]
Attal et al, 2017	205	350	169	350	19.4%	1.51 [1.12, 2.04]
Cavo et al, 2020	309	702	200	495	28.4%	1.16 [0.92, 1.46]
Gay et al, 2021	85	158	89	157	9.7%	0.89 [0.57, 1.39]
Richardson et al, 2022	171	365	150	357	19.9%	1.22 [0.91, 1.63]
**Subtotal (95% CI)**		**1843**		**1620**	**86.8%**	**1.22 [1.06, 1.41]**
Total events	820		647			
Heterogeneity: Tau² = 0.00; Chi² = 4.30, df = 5 (P = 0.51); I² = 0%						
Test for overall effect: Z = 2.82 (P = 0.005)						
**2.2.2 Observational studies**						
Hajek et al, 2018	31	236	63	741	9.2%	1.63 [1.03, 2.57]
Belotti et al, 2020	37	85	12	46	3.3%	2.18 [1.00, 4.79]
Bai et al, 2022	8	25	2	17	0.7%	3.53 [0.65, 19.28]
**Subtotal (95% CI)**		**346**		**804**	**13.2%**	**1.82 [1.24, 2.67]**
Total events	76		77			
Heterogeneity: Tau² = 0.00; Chi² = 1.03, df = 2 (P = 0.60); I² = 0%						
Test for overall effect: Z = 3.04 (P = 0.002)						
**Total (95% CI)**		**2189**		**2424**	**100.0%**	**1.29 [1.11, 1.49]**
Total events	896		724			
Heterogeneity: Tau² = 0.01; Chi² = 8.91, df = 8 (P = 0.35); I² = 10%						
Test for overall effect: Z = 3.43 (P = 0.0006)						
Test for subgroup differences: Chi² = 3.59, df = 1 (P = 0.06), I² = 72.1%						

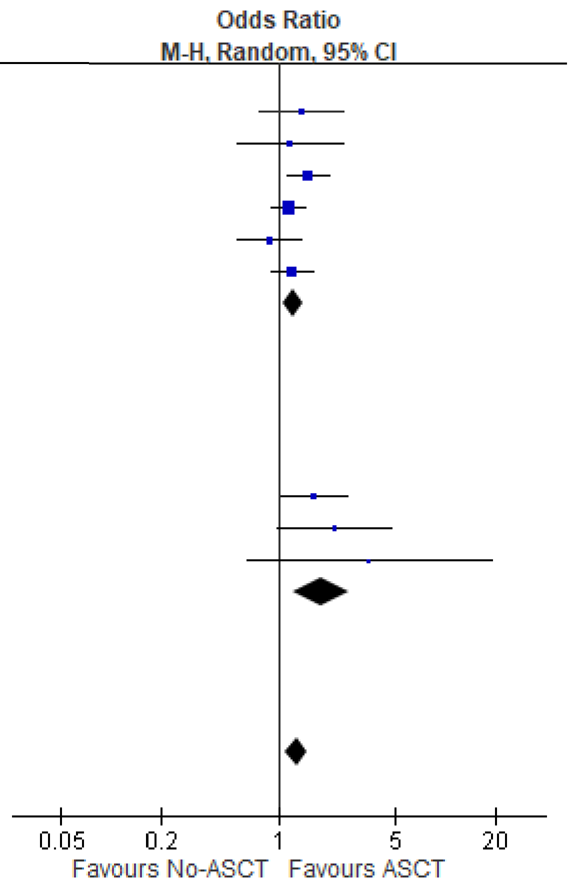

C. OR for CR (analysis 3)

Study or Subgroup	ASCT Events	ASCT Total	No-ASCT Events	No-ASCT Total	Weight	Odds Ratio M-H, Random, 95% CI
**2.3.1 RCTs (excluding 1 study)**						
Palumbo et al, 2014	33	141	24	132	5.9%	1.38 [0.76, 2.48]
Gay et al, 2015	17	127	15	129	3.7%	1.17 [0.56, 2.47]
Attal et al, 2017	205	350	169	350	21.5%	1.51 [1.12, 2.04]
Cavo et al, 2020	309	702	200	495	33.4%	1.16 [0.92, 1.46]
Gay et al, 2021	85	158	89	157	10.2%	0.89 [0.57, 1.39]
Richardson et al, 2022	171	365	150	357	22.1%	1.22 [0.91, 1.63]
**Subtotal (95% CI)**		**1843**		**1620**	**96.7%**	**1.22 [1.06, 1.41]**
Total events	820		647			
Heterogeneity: Tau² = 0.00; Chi² = 4.30, df = 5 (P = 0.51); I² = 0%						
Test for overall effect: Z = 2.82 (P = 0.005)						
**2.3.2 Observational studies (excluding 2 studies)**						
Belotti et al, 2020	37	85	12	46	3.3%	2.18 [1.00, 4.79]
**Subtotal (95% CI)**		**85**		**46**	**3.3%**	**2.18 [1.00, 4.79]**
Total events	37		12			
Heterogeneity: Not applicable						
Test for overall effect: Z = 1.95 (P = 0.05)						
**Total (95% CI)**		**1928**		**1666**	**100.0%**	**1.25 [1.08, 1.44]**
Total events	857		659			
Heterogeneity: Tau² = 0.00; Chi² = 6.33, df = 6 (P = 0.39); I² = 5%						
Test for overall effect: Z = 2.99 (P = 0.003)						
Test for subgroup differences: Chi² = 2.03, df = 1 (P = 0.15), I² = 50.7%						

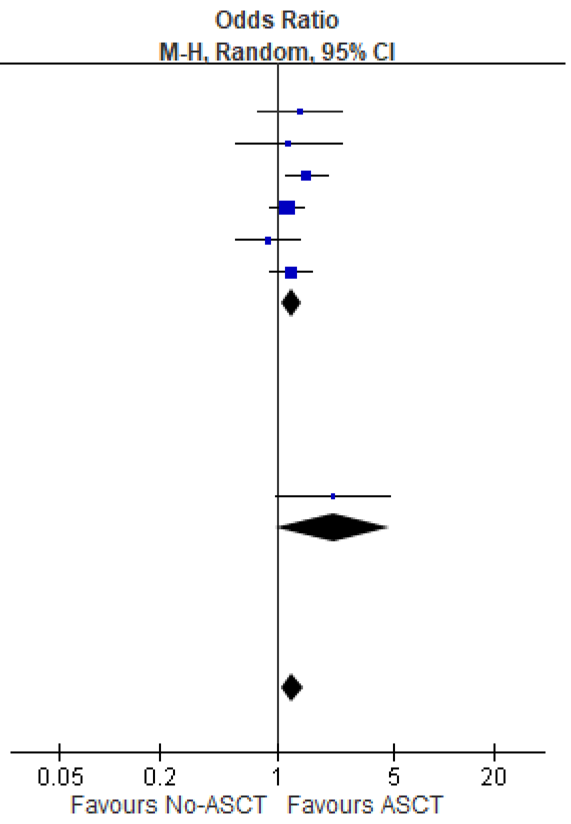

D. HR for PFS

Study or Subgroup	log[Hazard Ratio]	SE	ASCT Total	No-ASCT Total	Weight	Hazard Ratio IV, Random, 95% CI
**2.2.1 RCTs**						
Palumbo et al, 2014	-0.821	0.1625	141	132	9.3%	0.44 [0.32, 0.61]
Gay et al, 2015	-0.9203	0.2301	127	129	7.4%	0.40 [0.25, 0.63]
Attal et al, 2017	-0.4308	0.1041	350	350	10.9%	0.65 [0.53, 0.80]
Cavo et al, 2020	-0.3147	0.0833	702	495	11.4%	0.73 [0.62, 0.86]
Gay et al, 2021	-0.4943	0.1784	158	157	8.8%	0.61 [0.43, 0.87]
Richardson et al, 2022	-0.4308	0.1139	365	357	10.6%	0.65 [0.52, 0.81]
Yong et al, 2023	-0.3011	0.0986	109	109	11.0%	0.74 [0.61, 0.90]
**Subtotal (95% CI)**			**1952**	**1729**	**69.5%**	**0.62 [0.54, 0.72]**
Heterogeneity: Tau² = 0.02; Chi² = 14.06, df = 6 (P = 0.03); I² = 57%						
Test for overall effect: Z = 6.49 (P < 0.00001)						
**2.2.2 Observational studies (excluding 3 studies)**						
Cohen et al, 2018	-1.3863	0.3929	34	26	4.2%	0.25 [0.12, 0.54]
Belotti et al, 2020	-0.0943	0.3481	85	46	4.9%	0.91 [0.46, 1.80]
Lemieux et al, 2021	-1.204	0.3537	38	41	4.8%	0.30 [0.15, 0.60]
Cho et al, 2022	-1.5141	0.3093	54	156	5.6%	0.22 [0.12, 0.40]
Pawlyn et al, 2022	-0.8916	0.0955	404	366	11.1%	0.41 [0.34, 0.49]
**Subtotal (95% CI)**			**615**	**635**	**30.5%**	**0.37 [0.24, 0.55]**
Heterogeneity: Tau² = 0.13; Chi² = 11.44, df = 4 (P = 0.02); I² = 65%						
Test for overall effect: Z = 4.80 (P < 0.00001)						
**Total (95% CI)**			**2567**	**2364**	**100.0%**	**0.52 [0.43, 0.63]**
Heterogeneity: Tau² = 0.08; Chi² = 54.25, df = 11 (P < 0.00001); I² = 80%						
Test for overall effect: Z = 6.63 (P < 0.00001)						
Test for subgroup differences: Chi² = 5.76, df = 1 (P = 0.02), I² = 82.6%						

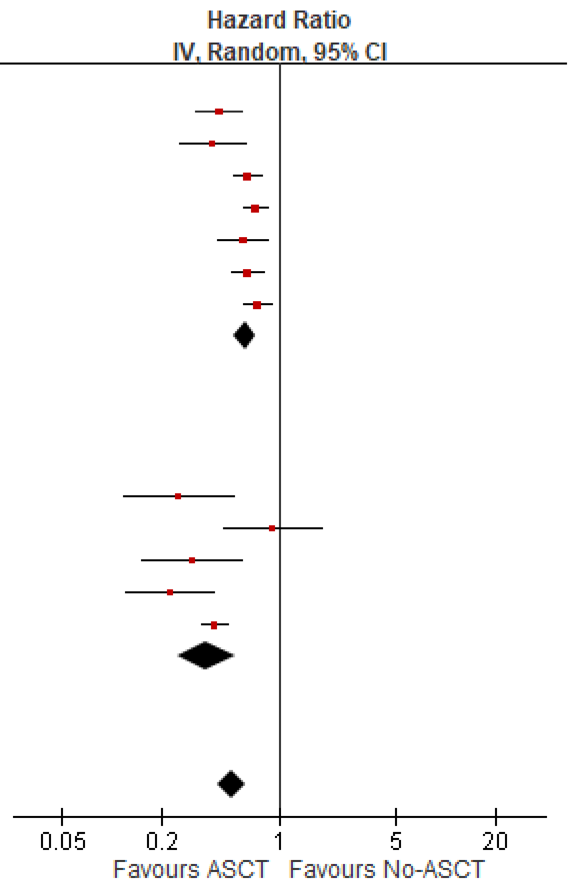

E. HR for OS

Study or Subgroup	log[Hazard Ratio]	SE	ASCT Total	No-ASCT Total	Weight	Hazard Ratio IV, Random, 95% CI
**2.3.1 RCTs**						
Palumbo et al, 2014	-0.5978	0.2763	141	132	5.9%	0.55 [0.32, 0.95]
Gay et al, 2015	-0.8754	0.307	127	129	5.1%	0.42 [0.23, 0.76]
Attal et al, 2017	0.1484	0.1896	350	350	8.9%	1.16 [0.80, 1.68]
Cavo et al, 2020	-0.1054	0.121	702	495	12.2%	0.90 [0.71, 1.14]
Gay et al, 2021	-0.0619	0.2828	158	157	5.7%	0.94 [0.54, 1.64]
Richardson et al, 2022	-0.0943	0.2041	365	357	8.3%	0.91 [0.61, 1.36]
**Subtotal (95% CI)**			**1843**	**1620**	**46.0%**	**0.82 [0.63, 1.05]**
Heterogeneity: Tau² = 0.05; Chi² = 10.99, df = 5 (P = 0.05); I² = 55%						
Test for overall effect: Z = 1.56 (P = 0.12)						
**2.3.2 Observational studies (excluding 7 studies)**						
Wildes et al, 2015	-0.6539	0.2806	62	84	5.7%	0.52 [0.30, 0.90]
Biran et al, 2016	-0.3425	0.1997	90	341	8.5%	0.71 [0.48, 1.05]
Cohen et al, 2018	-1.772	0.6349	34	26	1.6%	0.17 [0.05, 0.59]
Rosenberg et al, 2019	-0.3567	0.0378	2125	3184	15.9%	0.70 [0.65, 0.75]
Kaur et al, 2021	-0.5621	0.2069	378	561	8.2%	0.57 [0.38, 0.86]
Cho et al, 2022	-0.6349	0.644	54	156	1.5%	0.53 [0.15, 1.87]
Pawlyn et al, 2022	-0.6733	0.1114	404	366	12.7%	0.51 [0.41, 0.63]
**Subtotal (95% CI)**			**3147**	**4718**	**54.0%**	**0.59 [0.48, 0.72]**
Heterogeneity: Tau² = 0.03; Chi² = 13.65, df = 6 (P = 0.03); I² = 56%						
Test for overall effect: Z = 5.23 (P < 0.00001)						
**Total (95% CI)**			**4990**	**6338**	**100.0%**	**0.68 [0.58, 0.80]**
Heterogeneity: Tau² = 0.04; Chi² = 33.08, df = 12 (P = 0.0009); I² = 64%						
Test for overall effect: Z = 4.59 (P < 0.00001)						
Test for subgroup differences: Chi² = 3.91, df = 1 (P = 0.05), I² = 74.4%						

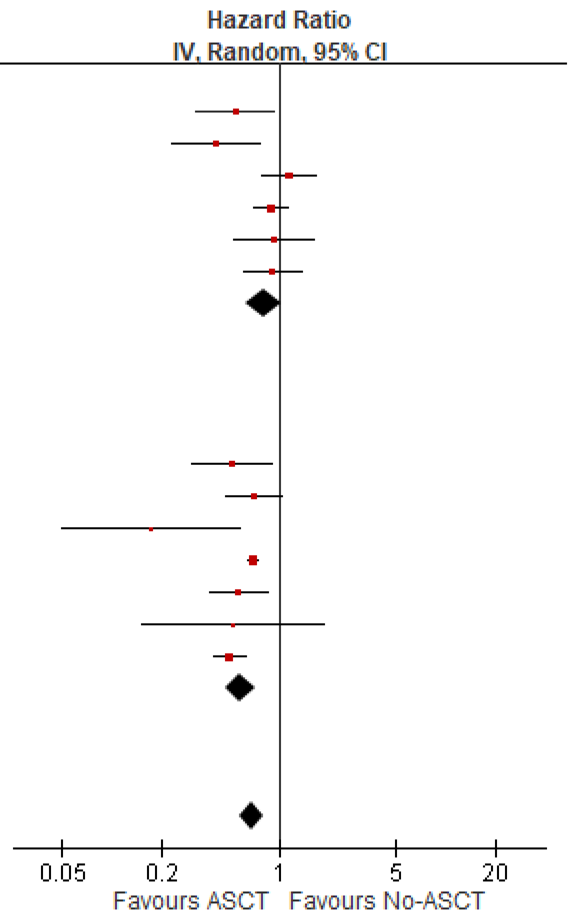

Supplementary Figure S1. Forest plots of the meta-analysis comparing ASCT and No-ASCT, excluding the studies with a serious risk of bias or a specialized design. A. OR of CR (analysis 1) excluding the studies with a serious risk of bias; B. OR of CR (analysis 2) excluding the studies with a specialized design; C. OR of CR (analysis 3) excluding the studies with a serious risk of bias and a specialized design; D. HR of mortality for PFS; E. HR of mortality for OS. RCT, randomized controlled trial; ASCT, autologous stem-cell transplantation; CR, complete response; PFS, progression-free survival; OS, overall survival; OR, odds ratio; HR, hazard ratio; M-H, Mantel-Haenszel method; IV, Inverse variance; SE, standard error; CI, confidence interval.

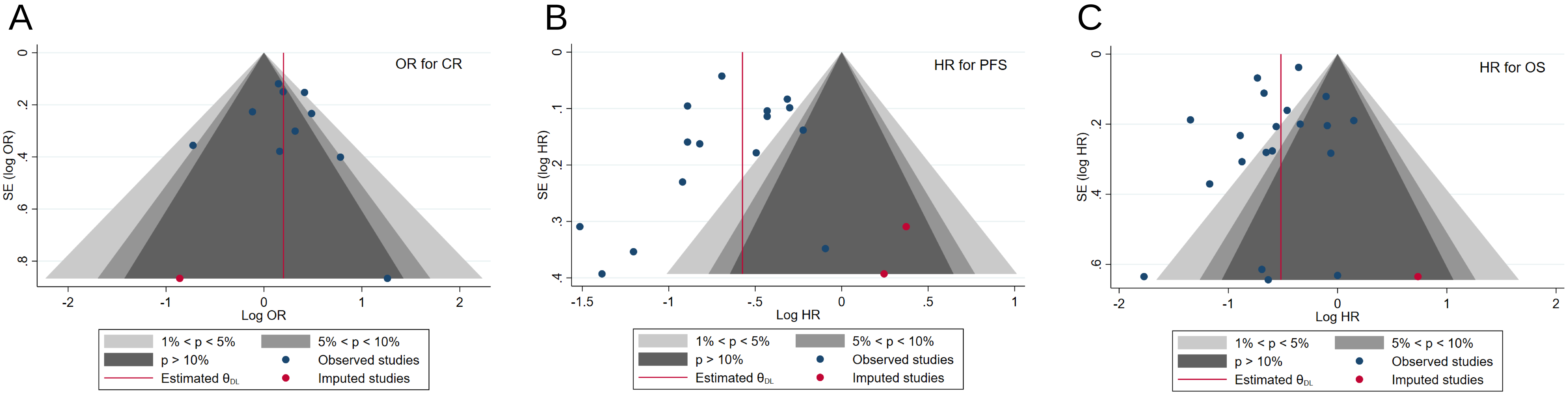

Supplementary Figure S2. Contour funnel plots of trim and fill method. A: OR for CR; B: HR for PFS; C: HR for OS; CR, complete response; OR. odds ratio; HR, hazard ratio; PFS, progression-free survival; OS, overall survival; SE, standard error.
